# Rasch analysis and targeting assessment of the teach-CVI survey tool in a cohort of CVI patients

**DOI:** 10.3389/fopht.2024.1495000

**Published:** 2024-11-29

**Authors:** Jem Martin, Chris Bradley, Barry S. Kran, Nicole C. Ross

**Affiliations:** ^1^ Department of Specialty, Advanced Care and Vision Science, New England College of Optometry, Boston, MA, United States; ^2^ Wilmer Eye Institute, Johns Hopkins School of Medicine, Baltimore, MD, United States; ^3^ Department of Psychology, Northeastern University, Boston, MA, United States

**Keywords:** Rasch analysis, cerebral visual impairment, cortical visual impairment, visual development, visual function, visually impaired children

## Abstract

**Purpose:**

Cerebral Visual Impairment (CVI) is the leading cause of pediatric visual impairment. Given the diversity of clinical presentations of CVI, we are interested in whether questionnaires appropriately target the spectrum CVI cases, specifically the Teach-CVI Screening Tool. Rasch analysis is a standard psychometric technique for assessing the targeting of questionnaire items, however this analysis technique has not yet been applied to this questionnaire.

**Methods:**

We performed a retrospective review of clinical CVI cases from the NECO Center for Eye Care at Perkins School for the Blind from January 2016 to December 2022. Electronic medical records were reviewed to identify patients with an ICD-9 or ICD-10 code of CVI or other neurological visual impairment. Age, gender, diagnoses, visual acuity, contrast sensitivity, visual fields, ocular alignment, and Teach-CVI responses were collected. We applied the method of successive dichotomizations, a polytomous Rasch model, to estimate item measures and person measures from the survey. Targeting of questionnaire items to the sample population was explored by comparing estimated item measures to person measures. Multiple linear regression was used to determine which factors influence patient visual ability (i.e., Teach-CVI person measure).

**Results:**

119 patient records were included, 54% of which were male. The mean age was 8.9 years (SD = 6.12) with a range of 0 to 33 years of age. Mean visual acuity was 0.46 logMAR (SD = 0.40), or 20/57. The majority of patients in the sample had a co-occurring visual disorder in addition to CVI (84%), the most frequent being strabismus (69.9%) or visual field loss (25.3%). Item measures ranged from -2.67 to 1.77 logits (SD = 0.76), with a mean of 0 logit by convention. Estimated person measures ranged from -2.19 to 3.08 logits (SD = 1.10) with a mean of -0.03 logit. The range of item measures covered 93.3% of the person measures, and all person measures, except one, were within one logit of an item measure. Visual measures were not statistically significantly associated with Teach-CVI person measures.

**Conclusion:**

The findings from this study suggest that the Teach-CVI survey is well targeted and an appropriate patient reported outcome measure for CVI.

## Introduction

Cerebral Visual Impairment (CVI) is the leading cause of visual impairment in children in developed countries (1–3). CVI is currently defined as “a verifiable visual dysfunction, which cannot be attributed to disorders of the anterior visual pathways or any potentially co-occurring ocular impairment” (4, 5).

While some impairments may be found in the primary visual cortex, CVI is characterized by deficits in visual processing beyond the primary visual cortex (5, 6). These deficits are not easily detected through routine eye examinations, which has led to the development of instruments – some are used to guide the clinical interview (7, 8) while others are used as screening tools to screen for symptoms potentially related to CVI (9, 10).

One such instrument is the Teach-CVI screening tool (11). Created by the Teach-CVI partnership, Teach-CVI has three different levels – three different sets of questions (or items) regarding the frequency of behaviors known to be related to CVI for a given developmental age and/or motor ability. Level 1 has 19 items and is intended for those who are non-ambulatory. Level 2 has 35 items and is intended for individuals with a developmental age between 2 and 6 years old. Level 3 has 45 items and is intended for individuals with a developmental age of between 6 and 12 years old. Responses are on a 4-point Likert scale: (1) never, (2) occasionally, (3) frequently, and (4) always.

Teach-CVI responses are typically analyzed by tallying the number of times a person responded with a 3 or a 4, i.e. a “positive score” – for some items the scale is reversed (i.e., a “positive score” corresponds to a response of 1 or 2) and must be flipped prior to analysis. Analyzing scores in this way is problematic for two reasons. First, a Likert scale is not necessarily an equal interval scale. For example, the difference between 1 and 2 on a Likert scale does not *a priori* represent the same difference in latent trait (i.e., functional vision in the case of Teach-CVI) as the difference between 2 and 3, or between 3 and 4. Furthermore, dichotomizing responses into “positive” and “non-positive” does not utilize the full 4-point Likert scale.

Rasch analysis is a standard psychometric technique used to convert Likert scale responses into an equal interval scale. Rasch analysis estimates item measures and person measures on the same measurement scale, allowing them to be directly compared – item measures represent the magnitude of the underlying latent trait required by the item while person measures represent the magnitude of the latent trait possessed by the person (see (12) for further description of item and person measures in low vision rehabilitation, and (13) for further discussion of measurement with Rasch analysis). Both item and person measures are calculated in units of logit, whereby higher item measures indicate more difficult items, and higher person measures represent having more of the latent trait being measured. In our application, with Teach-CVI, this trait is visual ability. Unlike raw scores or sums or means of raw scores, Rasch analysis estimates person and items measures on the same equal interval scale despite missing data for some person-item combinations.

Rasch analysis has become the preferred psychometric technique for analysis of patient reported outcome questionnaire data in many fields, including low vision rehabilitation (12, 14–16). One reason is because Rasch analysis allows assessment of targeting – how precisely a set of questionnaire items can measure the latent trait in the sample of persons. Mathematically, targeting is assessed by comparing the item measure distribution to the person measure distribution, which ideally should be similar. Targeting is important because person measures can be more precisely estimated the closer they are to item measures. A questionnaire that is not well targeted may have ceiling or floor effects – person measures far above the item measure distribution (a ceiling effect) or person measures far below it (a floor effect), and thus may not be suitable for measuring the latent trait in a particular patient sample. The problem with ceiling and floor effects is that it becomes difficult to discriminate between person measures near the ceiling or the floor. Targeting should be assessed during the development of any questionnaire that uses Likert scales (16). In some instances, questionnaires were widely adopted and utilized before ceiling or floor effects were discovered, making it more difficult to modify the questionnaire afterwards (14, 17–24).

Another psychometric property of questionnaires that should be evaluated is unidimensionality, i.e., whether or not all items measure the same construct (same latent trait). A set of items exhibits unidimensionality if the variance in responses to the items can be explained by one source, the latent variable. Factor analysis has been a common method of assessing the dimensionality of questionnaires, in which the number of factors that explain the variance in responses can be explored. Rasch analysis can also be used to assess unidimensionality through its infit mean square statistic (infit).

In this study, we apply Rasch analysis to Teach-CVI to assess targeting and unidimensionality in a sample of patients with a diagnosis of CVI. This psychometric analysis is necessary to determine if Teach-CVI is potentially suitable for use as a patient-reported outcome measure in CVI patients, in addition to its intended use as a screening tool. We also explore how demographic variables and clinical visual function measures (e.g., visual acuity, visual field and contrast sensitivity) influence the latent trait of visual ability as measured by Teach-CVI person measures.

## Methods

Teach-CVI responses were retrospectively analyzed from patients with a diagnosis of CVI that presented to the NECO Center for Eye Care at Perkins School for the Blind, a tertiary clinic serving children with low vision and multiple impairments, from January 2016 to December 2022. Charts were identified through searching by ICD-10 codes for cortical blindness, and common etiologies and comorbidities of CVI such as periventricular leukomalacia and hypoxic ischemic encephalopathy. The list of ICD-10 codes searched included all of the following: H47.61X “Cortical blindness”, P91.2 “Periventricular leukomalacia”, P91.60 “Hypoxic ischemic encephalopathy.” The ICD-9 code for cortical blindness (377.75) was also used. A total of 183 charts fit the search criteria. Of those, 136 had at least one completed Teach-CVI, and of those 119 had a diagnosis of CVI and were included in our study.

Demographic information (age, gender, neurologic history and comorbid diagnoses) and visual measures (visual acuities, contrast sensitivity, presence of strabismus, presence of visual field defect) from the most recent vision examination were collected, as well as Teach-CVI responses. While the Teach-CVI tool has been highly utilized at the NECO Center for Eye Care at Perkins School for the Blind, only those patients for which the complete raw data Teach-CVI forms were available were included. Charts with only summary score information were excluded. In cases where multiple surveys were completed for the same patient, the survey completed most recently by a parent was selected. Those without a diagnosis of CVI were excluded.

Examination approaches varied between patients in order to allow for the maximum engagement from the patient, meaning that testing methods for clinical vision measures were not consistent between patients. Distance visual acuity tests included various electronically displayed formats as well as the Feinbloom chart. Near acuity tests included various near cards as well as recognition tests (such as Teller Cards). Qualitative assessment of acuity included fix and follow and light perception, and had their visual acuity reported as a missing variable. Contrast sensitivity testing utilized the Double Happy cards (25), Pelli Robson (26), Berkeley Blinking Squares (27–29) and MARS cards (30). **Table 1** reports the type of visual acuity charts and contrast sensitivity tests used in our sample of CVI patients.

**Table 1:** Clinical tests of visual acuity and contrast sensitivity utilized.

Visual Acuity Test	n (%)
Snellen acuity	38 (31.93%)
Patti pics/Lea symbols	27 (22.69%)
HOTV	7 (5.88%)
ETDRS	5 (4.20%)
FrACT*	2 (1.68%)
Feinbloom	1 (0.84%)
Teller Acuity Cards	35 (29.42%)
Missing	4 (3.36%)
Contrast Sensitivity Test	
Pelli-Robson Card	31 (26.05%)
MARS	3 (2.52%)
Double Happy Cards	76 (63.87%)
Berkeley Blinking Squares Contrast App**	4 (3.36%)
Missing	5 (4.20%)

*The Freidberg Visual Acuity Test (FrACT) digital visual acuity test, (31, 32), https://michaelbach.de/fract/

**Berkeley Blinking Squares Contrast app (27–29); https://apps.apple.com/us/app/berkeley-contrast-squares/id979063261

This study was reviewed and approved by the institutional review board (IRB) at the New England College of Optometry and adhered to the tenets of the Declaration of Helsinki. Requirement for informed consent was waived by the IRB due to the retrospective nature of the study.

### Teach-CVI

Teach-CVI has three different levels (i.e., different sets of questionnaire items) that can be administered based on mobility and/or developmental age. The level chosen for completion was determined by the provider at the time of the examination after review of the patient’s medical, ocular, and educational records, and after time spent with the patient during the examination.

Survey items are asked on both an ability and disability scale (e.g. “Makes eye contact” is on an ability scale, while “Has difficulties with looking at objects” is on a disability scale). For the purposes of the analysis, responses to items using a disability scale were “flipped” to lie on an ability scale for consistency across items.

### Rasch analysis

Teach-CVI responses were analyzed using the method of successive dichotomizations (MSD), which is a polytomous Rasch model that estimates person and item measures on the same equal interval scale regardless of the number of response categories, an improvement over previous Rasch models (33). The R package ‘msd’ was used for analysis. Responses from all three Teach-CVI levels were simultaneously analyzed, so that items common to different Teach-CVI levels had their item measures estimated from all participants who responded to those items.

To assess targeting the distributions of estimated person and item measures were compared to each other. Ideally, the two distributions are similar so that more items are available to precisely estimate the majority of person measures. To confirm that Teach-CVI measures a single latent trait, we assessed unidimensionality through the person and item infit mean square statistics (infits). The infit compares the observed variance in the responses to the expected, which is 1 if the unidimensionality assumption in Rasch models is satisfied. Most person and item infits should lie between 0.5 and 1.5 for evidence of unidimensionality (34).

### Statistical analysis

Multilinear regression was used to determine the influence of different variables on the estimated person measures. These variables were: demographic information (age, gender), clinical visual function (e.g., visual acuity, contrast sensitivity, presence of visual field deficit, presence of strabismus), neurologic diagnoses categorized according to prevalence rates in CVI (35) (specifically, categorized as: “gestational and perinatal inflammation” including periventricular leukomalacia, hypoxic ischemic encephalopathy, hypoxia/anoxia, stroke, hydrocephaly, and meningitis; genetic/metabolic conditions, traumatic, and “other”) and level of impairment (categorized as: diagnosis of CVI only, CVI and other visual diagnoses, CVI and co-occurring physical impairment (e.g., cerebral palsy, hemiplegia), or multiply disabled with CVI and both a visual and physical disability).

## Results

**Table 2** shows demographic and clinical information of our sample. A total of 119 patient electronic health records were included in the review, 54% of which were male. The mean age was 8.9 years (SD = 6.12) with a range of 0 to 33 years of age. Mean visual acuity was 0.46 logMAR (SD = 0.40), or 20/57, with a range from 20/10 to 20/1,9000 Snellen equivalent. The majority of patients, 48%, had multiple impairments with both visual and physical impairments in addition to CVI. Those with a diagnosis of CVI and another visual diagnosis only made up 36% of the sample, and 6% had a physical disability only in addition to CVI. The most frequent co-existing visual condition was strabismus (69.9%), and the next most common ocular/brain based visual function finding was the presence of a visual field deficit (25.3%). The range of co-occurring ocular and binocular pathologies that were noted included: nystagmus, optic atrophy, binocular vision dysfunction, cranial nerve palsies, retinal conditions including retinopathy of prematurity, retinitis pigmentosa, colobomas, and other specific non-refractive conditions.

**Table 2:** Demographics and clinical information.

	Mean (SD)
Age	9.18 years (6.07)
Visual Acuity	0.46 logMAR (0.40)
Contrast Sensitivity	1.57 logCS (0.46)
	N (%)
Female	54 (45.38%)
Male	65 (54.62%)
Strabismus	83 (69.75%)
Visual field defect*	30 (25.21%)
Teach-CVI Level 1	21 (17.65%)
Teach-CVI Level 2	51 (42.86%)
Teach-CVI Level 3	47 (39.50%)
Visual impairment**	44 (36.97%)
Physical impairment**	7 (5.88%)
Multiply impaired**	57 (47.90%)
CVI only**	11 (9.24%)

*Presence of any visual field loss.

**Visual impairment refers to diagnosis of CVI and another visual diagnosis only, physical impairment for CVI and a physical diagnosis, multiply impaired if the diagnoses included CVI, visual, and physical impairments.

**Figure 1:**
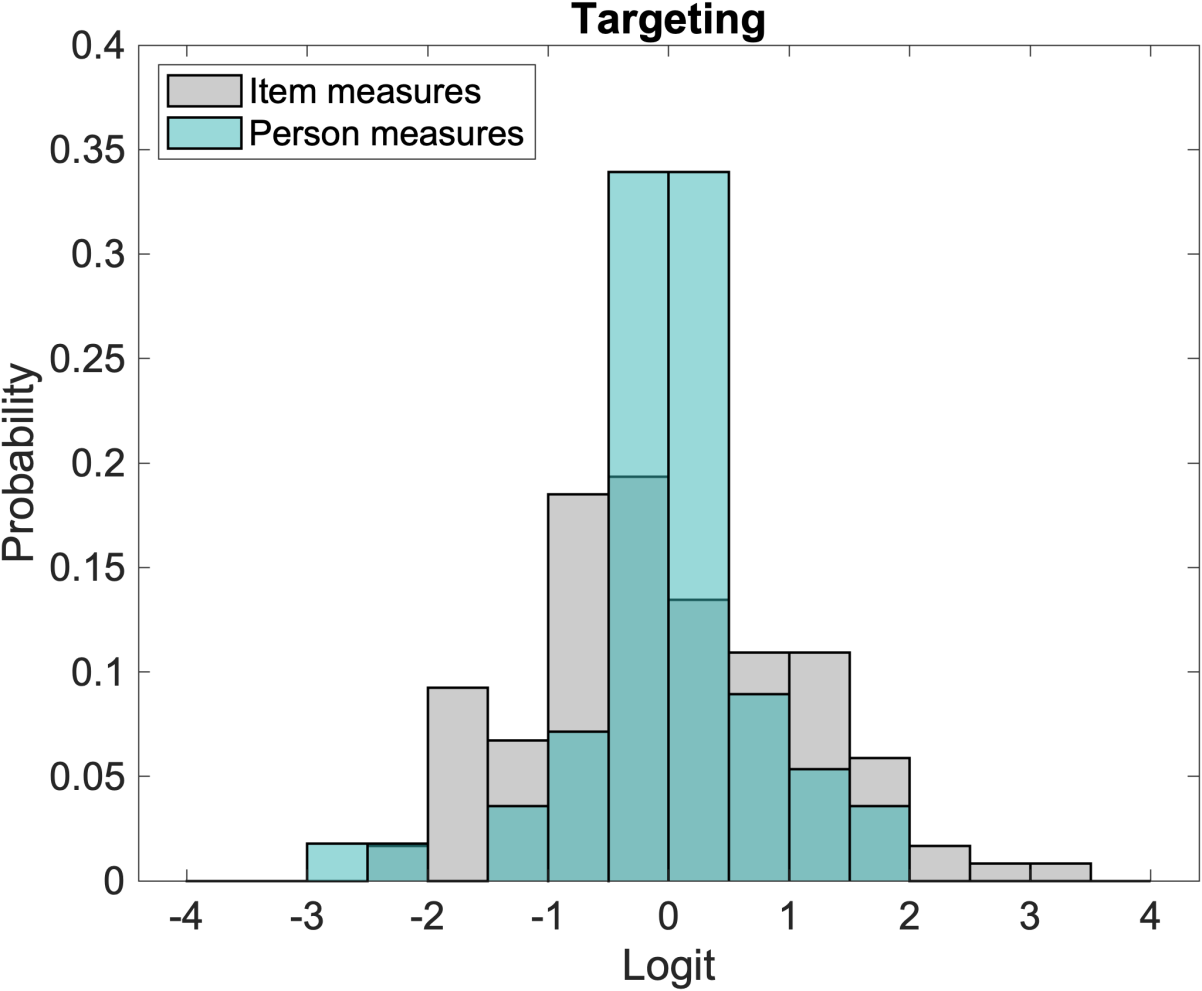
Comparison of the distribution of person measures (teal bars) and item measures (grey bars).

**Figure 1** compares the distribution of estimated person measures (teal) against the distribution of item measures (grey). Item measures ranged from -2.67 to 1.77 logits (SD = 0.76), with a mean of 0 logit by convention. Estimated person measures ranged from -2.19 to 3.08 logits (SD = 1.10) with a mean of -0.03 logit. The range of item measures covered 93.3% of the person measures, and all person measures, except one, were within one logit of an item measure. This provides evidence that Teach-CVI is relatively well targeted to our CVI patient sample.

**Figure 2:**
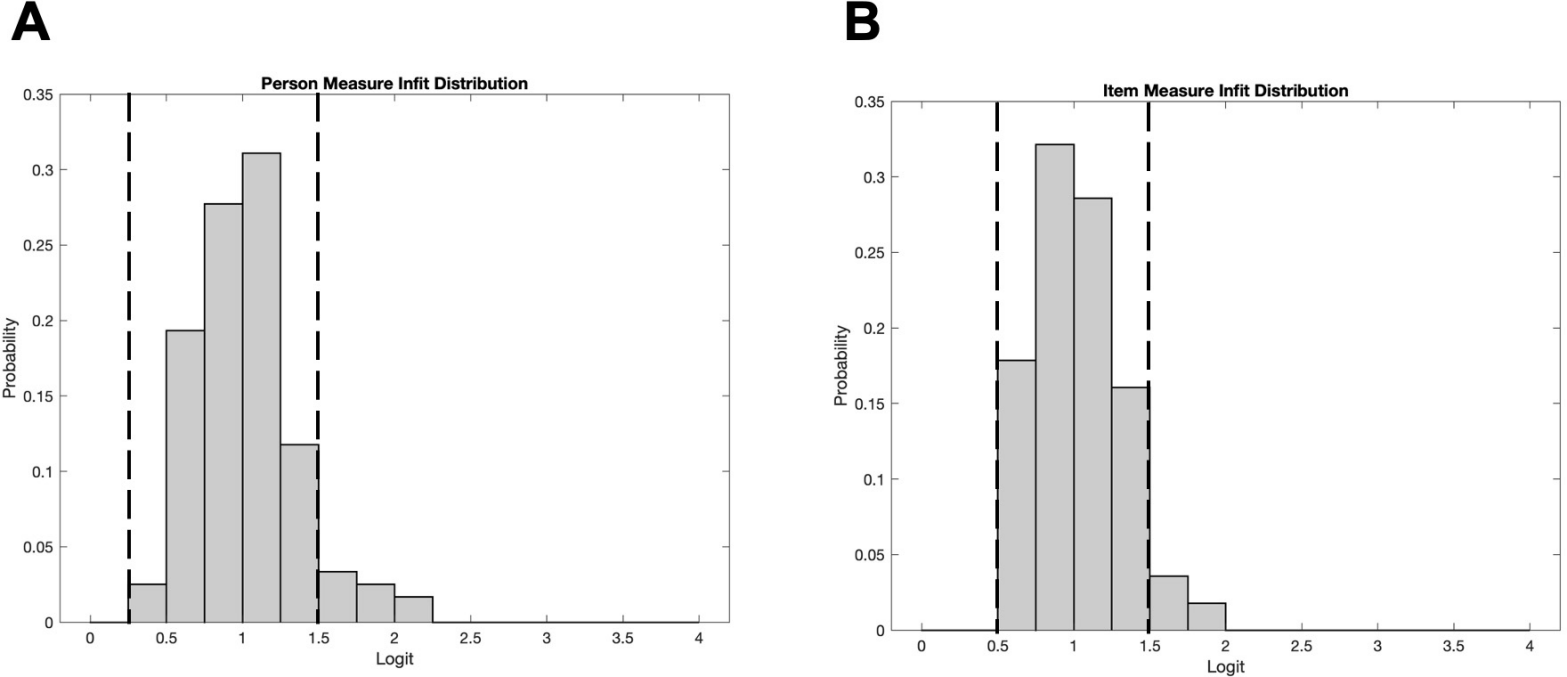
**(A)** Distribution of item measure infits. **(B)** Distribution of person measure infits. Unidimensionality is demonstrated when the data largely falls between 0.5 and 1.5 (indicated by the vertical dashed lines).

**Figure 2** illustrates the infits for item measures and person measures. Item measure infits ranged from 0.60 to 1.85 with a mean (SD) of 1.03 (0.28). Person measure infits ranged from 0.20 to 2.24 with a mean (SD) of 1.02 (0.34). Ideally, the mean infit is 1. Approximately 95% of item measure infits and 90% of person measure infits fell within the desired range of 0.5-1.5 (34) which are indicated by the vertical dashed lines. The person and item infit distributions provide evidence that Teach-CVI measures a single underlying construct (i.e., the instrument is unidimensional).

**Figure 3:**
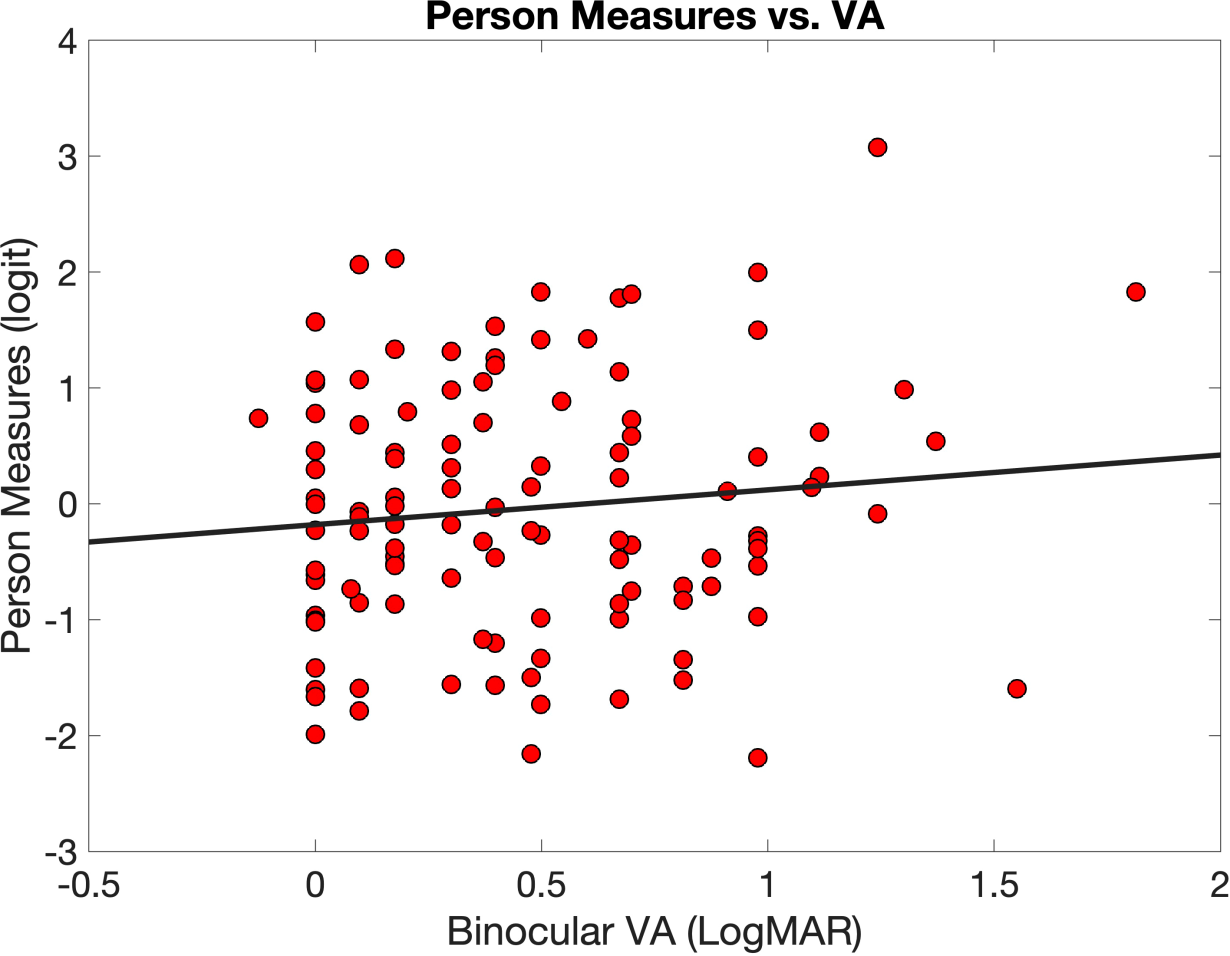
Person measures (red circles) plotted against logMAR binocular visual acuity (VA). The equation for the linear regression line (black line) is y = 0.3x−0.18 .

**Figure 3** illustrates the relationship between person measures (y-axis) and visual acuity (x-axis).

The correlation was r = 0.011, and the equation of the best fitting line is y = 0.3x−0.18 (the slope of 0.3 was not significant at p = 0.24). In other words, there is no evidence of a significant relationship between Teach-CVI-person measures and visual acuity in our sample.

**Table 3** shows the results of multilinear regression to determine the influence of other demographic, visual, and neurologic variables on Teach-CVI person measures. The reference groups were: CVI only for impairment category, and history of gestational or perinatal inflammation (including PVL, HIE, hypoxia, meningitis, hydrocephaly) for neurologic history. None of the included variables were found to be significantly associated with the Teach-CVI person measures.

**Table 3:** Multilinear regression of potential associated factors with Teach-CVI person measures.

	Estimate	Standard Error	t value	Pr(>|t|)
(Intercept)	-0.04	0.56	0.07	0.94
Age	0.01	0.02	0.65	0.51
Contrast Sensitivity (logCS)	-0.17	0.25	-0.68	0.50
Gender: Male	-0.21	0.21	-0.99	0.33
Impairment Category: Visual Impairment	0.40	0.43	0.93	0.35
Impairment Category: Physical Impairment	-0.58	0.57	-1.02	0.31
Impairment Category: Multiply Impaired	0.38	0.45	0.85	0.40
Visual Field Loss	0.09	0.25	0.35	0.73
Presence of strabismus	-0.05	0.28	0.17	0.87
Neurologic History: Genetic/Metabolic	0.30	0.46	0.65	0.52
Neurologic history: Traumatic	0.39	0.67	0.58	0.57
Neurologic history: Other (Prematurity, Epilepsy/seizure disorder)	-0.03	0.25	-0.13	0.90

## Discussion

This study is the first to explore the psychometric properties of the Teach-CVI instrument through the application of Rasch analysis, offering a novel approach to evaluating visual ability in patients with Cerebral Visual Impairment (CVI). Our findings demonstrate that Teach-CVI is well-targeted to the CVI patient sample, as evidenced by the alignment of estimated person and item measures. The infit statistics provide evidence for unidimensionality of the instrument, which is critical for ensuring that Teach-CVI accurately measures a single construct: the impact of CVI on visual ability.

As anticipated, primary visual cortex (V1) measures of visual function—visual acuity, visual field, and contrast sensitivity—were not statistically significant predictors of Teach-CVI person measures. This finding is consistent with clinical observations that patients with CVI can often retain good visual acuity but experience profound functional difficulties (5, 6, 36, 37). The disconnect between visual acuity and visual function in CVI has been documented in clinical practice (38, 39), but our analysis provides a more definitive demonstration of this phenomenon. Although visual field defects may be related to occipital lobe damage and therefore can be attributed to CVI, our sample of patients included both ocular based and neurologically based field loss, and therefore was included as a variable to explore whether person measures may correlate with visual field loss independently. While some patients in our sample presented with 20/20 (0.0 logMAR) acuity and full visual fields, they still reported significant functional impairments, underscoring the importance of tools like Teach-CVI in capturing the true extent of CVI’s impact on visual ability. This absence of a relationship provides support for the construct validity of Teach-CVI, as CVI often presents with preserved visual acuity despite significant visual processing deficits. Further, neither neurologic history nor other additional impairments (e.g., physical/motor) were significant, further underscoring CVI’s ability to have profound impacts on the individual’s functional use of vision regardless of etiology or co-occurring diagnoses.

Other CVI instruments like the Top 11 (6), CVI Questionnaire (40), The Five Questions (10), Dutton CVI Inventory (7), and Paediatric Evaluation of Disability Inventory, Dutch version (41) have been employed for screening and diagnostic purposes but have predominantly relied on raw scores for analyses. Raw score-based methods can be skewed by missing data – one person could respond to only “easy” items, while another could respond to only the “difficult” items, and obtain the same mean raw score. Rasch analysis, in contrast, is robust against missing data and offers more precise person measures. Missing data or “not applicable” responses to any person-item combinations will reduce the precision of the estimated person measure in Rasch analysis, but not the measure itself, with large enough sample size. This property of Rasch analysis is particularly important in the context of CVI, where the heterogeneity of patient experiences may lead to different response patterns.

Previous studies have used methods such as exploratory factor analysis (10, 40) to assess dimensionality, ROC sensitivity analysis to explore sensitivity and specificity for detecting CVI (9) and Cronbach’s alpha (10, 41) to assess internal consistency of CVI instruments. However, none of these approaches enable person and item measures to be estimated on the same equal interval scale. Cronbach’s alpha in particular is often misinterpreted and misused as a measure of internal consistency (42, 43).

Rasch analysis has numerous applications beyond psychometric validation. It can be used to compare person measures pre- and post-intervention, track longitudinal changes, and perform cross-cultural validation by comparing item measures across different populations. The ability to use the same set of (calibrated) item measures for different studies also enables person measures from different samples to be compared on the same measurement scale. Given its broad utility and widespread adoption in other healthcare sectors (44–51), it is strongly recommended that Rasch analysis be applied to other CVI questionnaires to thoroughly explore their psychometric properties, including targeting.

The Teach-CVI survey, with its flexible, individualized approach, is particularly well-suited for assessing patients across a spectrum of CVI severity. Although originally designed as a screening tool, our study employed Teach-CVI as a guided history, allowing for a deeper understanding of each patient’s visual ability. However, there are several limitations, most notably that this was a retrospective study with a sample of patients from a single tertiary clinic. Future studies should aim to include a larger sample from multiple clinics. Additionally, approaches to clinical testing of acuity, contrast, and other measures differed between subjects. However, this was necessary and done to better match the ability of the individual and provide the most accurate measure of their functional vision.

MRI data, which is often used in conjunction with clinical findings and observations to make a diagnosis of CVI, was not available for all patients. This is not unusual given the systemic comorbidities in this patient population. This study also did not further delineate between intellectual and developmental disabilities as this information was not always available to the practitioner. Future studies could implement additional clinical tests to more accurately determine developmental age and level of intellectual impairment or developmental delay, such that the impact of intellectual disability on Teach-CVI person measures could be further explored.

## Conclusion

This study represents an important step forward in validating the Teach-CVI screening tool for use as a patient reported outcome measure in patients with CVI. Our results confirm that Teach-CVI is a psychometrically valid instrument that is well-targeted to a sample of CVI patients, and capable of measuring functional vision independently of visual acuity. The application of Rasch analysis has allowed for a more nuanced understanding of the instrument’s performance, offering a significant improvement over the traditional raw score-based approach.

While our study is limited by its retrospective nature, small sample size, and variability in clinical testing approaches, it nonetheless introduces a robust method for evaluating CVI-related visual function. Future research should focus on increasing sample size, further refining the Teach-CVI tool, and applying Rasch analysis to other CVI questionnaires. Additionally, longitudinal studies tracking changes in person measures over time could provide valuable insights into the efficacy of interventions for CVI patients.

## Data Availability

The raw data supporting the conclusions of this article will be made available by the authors, without undue reservation.
